# Gene-Based Testing of Interactions Using XGBoost in Genome-Wide Association Studies

**DOI:** 10.3389/fcell.2021.801113

**Published:** 2021-12-16

**Authors:** Yingjie Guo, Chenxi Wu, Zhian Yuan, Yansu Wang, Zhen Liang, Yang Wang, Yi Zhang, Lei Xu

**Affiliations:** ^1^ Institute of Fundamental and Frontier Sciences, University of Electronic Science and Technology of China, Chengdu, China; ^2^ School of Electronic and Communication Engineering, Shenzhen Polytechnic, Shenzhen, China; ^3^ Department of Mathematics, University of Wisconsin-Madison, Madison, WI, United States; ^4^ Research Institute of Big Data Science and Industry, Shanxi University, Taiyuan, China; ^5^ School of Life Science, Shanxi University, Taiyuan, China; ^6^ Beidahuang Industry Group General Hospital, Harbin, China

**Keywords:** genome-wide association studies, gene–gene interactions, XGBoost, additive model, gene-based testing

## Abstract

Among the myriad of statistical methods that identify gene–gene interactions in the realm of qualitative genome-wide association studies, gene-based interactions are not only powerful statistically, but also they are interpretable biologically. However, they have limited statistical detection by making assumptions on the association between traits and single nucleotide polymorphisms. Thus, a gene-based method (GGInt-XGBoost) originated from XGBoost is proposed in this article. Assuming that log odds ratio of disease traits satisfies the additive relationship if the pair of genes had no interactions, the difference in error between the XGBoost model with and without additive constraint could indicate gene–gene interaction; we then used a permutation-based statistical test to assess this difference and to provide a statistical *p*-value to represent the significance of the interaction. Experimental results on both simulation and real data showed that our approach had superior performance than previous experiments to detect gene–gene interactions.

## 1 Introduction

Genome-wide association study (GWAS) is a collection of successful methods for identifying genetic loci associated with complex traits. More than 71,000 specific single nucleotide polymorphisms (SNPs) associated with diseases or traits have been identified (Hindorff et al., 2009; Yang et al., 2015; Liu et al., 2018a; Guo et al., 2018; Buniello et al., 2019; Loos, 2020; Lyu et al., 2020; Hu et al., 2021). Previous GWAS schemes relied mainly on a single locus model that verified the independent association of individual markers to particular phenotypes. Despite the successful recognition of many regions of disease susceptibility, most SNPs captured by this kind of method may have a small effect size that does not explain the heritability of complex traits fully. It is believed that genetic interactions that are engaged significantly in the genetic basis of complex traits and diseases (Cordell, 2009; Moore et al., 2010; Liu et al., 2018b; He et al., 2020; Luo et al., 2020; Shao and Liu, 2021) may be a potential solution to the problem of “missing heritability” (Manolio et al., 2009; Fang et al., 2019; Young, 2019). The solution may be partial, but it could enlighten the construction of new topologies for gene pathways.

Genetic interaction was first studied at the SNP level, and SNP–SNP interactions (i.e., epistasis) were detected by applying several methods (Li et al., 2015a; Ritchie and Van Steen, 2018), such as statistics based on entropy (Dong et al., 2008), logistic regression (Lin et al., 2016), and odds ratio (Emily, 2012); other techniques include multifactor dimensionality reduction (MDR) (Ritchie et al., 2003), BOOST (Wan et al., 2010), RRIntCC (Zhang et al., 2019), GenEpi (Chang et al., 2020), and some accelerate method (Nobre et al., 2021). One of the general challenges encountered by these SNP-based approaches is the statistical weakness of the higher-order or pairwise tests that result from massive multiple testing corrections over all the groups or pairs of SNPs. Instead, we investigated every possible SNP from two genes in single, gene-based interaction detection.

The success of gene-based approaches in marginal association studies of GWAS could extend to the analysis of gene–gene interactions (GGIs) (Emily, 2018; Emily et al., 2020). This approach has several potential advantages. First, it typically has far fewer genes than SNPs, reducing the number of pairwise tests drastically. For example, $∼2\times 10^{8}$ tests are required to detect genetic interactions in pairs of 20,000 genes. However, over $5\times 10^{12}$ tests are required for 3 million SNPs in a marker-based interaction. Second, because a gene contains more information than a single SNP and genes interact diversely, gene-based methods are more powerful statistically, which applies to gene-based studies on the main effects as well (Liu et al., 2010; Li et al., 2011; Jiang et al., 2017; Wang et al., 2020; Wang et al., 2021). Additionally, biological prior knowledge (e.g., information about the known association of genes within protein–protein interactions (PPIs) or pathways) can be introduced easily. Finally, gene-based results are characterized by having better interpretability and important biological consequences.

Peng et al. (Peng et al., 2010) discovered a canonical correlation of a pair of genes in a case group and in a control group by applying a canonical correlation analysis–based U statistic (CCU), which measured the difference in the correlation of the gene pair. The difference then indicated the incidence of a GGI. In the analysis, however, only linear relationships were taken into consideration. Afterward, CCU was extended to kernelized CCU (KCCU) (Yuan et al., 2012; Larson et al., 2013), where a non-linear relationship was detected under the kernel. Recently, Emily (Emily, 2016) presented a method called AGGrGATOr that combined *p*-values interaction tests at the marker level to gauge how a pair of genes interacted, which was a strategy used by Ma et al. (Ma et al., 2013) earlier to detect interactions under quantitative phenotypes. Li et al. (Li et al., 2015b) proposed an entropy-based and nonparametric method called GBIGM.

At present, the new approach GGInt-XGBoost is proposed for identifying gene–gene interactions of complex phenotypes at the gene level in case-control studies by leveraging the eXtreme Gradient Boosting (XGBoost) (Chen and Guestrin, 2016), which is applied in co-expressed gene detection and to explore genetic associations in the field of bioinformatics (Jiang et al., 2013; Babajide Mustapha and Saeed, 2016; Liu et al., 2016; Liu and Jiang, 2016; Mrozek et al., 2016; Wei et al., 2017a; Wei et al., 2017b; Liu et al., 2017; Chen et al., 2018; Wei et al., 2018; Jiang et al., 2019; Liu et al., 2019; Yu et al., 2020a; Yu et al., 2020b; Lv et al., 2020; Li et al., 2021; Liu et al., 2021). A built-in mechanism of XGBoost is that one can impose constraints on the trained model to make it additive, which we assume characterizes the lack of interaction between two genes. Our method exhibited an outstanding performance for detecting the underlying gene–gene interactions at the gene level under various settings based on the experiments using a semi-empirical dataset. Its application using real datasets showed accurate identification of gene–gene interactions.

## 2 Materials and Methods

In this section, we detailed the statistical workflow for GGInt-XGBoost. To evaluate the power to detect GGIs and type-I error, we present the different parameter settings for simulation studies based on empirical data. Then, we adopted a real-world rheumatoid arthritis dataset from the WTCCC (Wellcome Trust Case–Control Consortium) database to assess the performance of our method under a real situation.

### 2.1 GGInt-XGBoost

#### 2.1.1 Preliminaries and Notation

Here, we take genes, a couple of SNPs, as the basic unit. Suppose that we have n random samples:

$$\left(G_{1,i},G_{2,i}\right)\in {ℛ}^{p+q},i=1,2,\ldots ,n,$$ (1)

where

$$G_{1,i}=\left(g_{1,i,1},g_{1,i,2},\ldots ,g_{1,i,p}\right),G_{2,i}=\left(g_{2,i,1},g_{2,i,2},\ldots ,g_{2,i,q}\right),\;i=1,2,\ldots ,n$$

and $G_{1}$ and $G_{2}$ represent two genes each with p and q SNPs, independently. In the case–control studies, $y_{i}\in \left\{0,1\right\}$ is a categorical label, where 0 is a control subject and 1 is a case subject. $g_{k,i,j}\in \left\{0,1,2\right\}$ represents the copy number of the minor alleles of SNP j in the gene k for the sample i .

In this work, we created a statistic based on the XGBoost to quantify GGI intensity in order to see if there is a statistical interaction between two genes in a qualitative phenotype. To estimate the distribution of the statistic, we used a permutation resampling strategy. Our method was based on the assumption that if there was no interaction between two genes, adding a constraint to limit interactions between SNPs to only occurring in the same gene would not have a significant negative impact on XGBoost performance. The XGBoost’s build-in mechanism for adding interaction constraints enables us to generate an additive model and use prior knowledge about the gene structure during model construction.

#### 2.1.2 Definition of Total Additivity for Gene–Gene Interaction

We defined GGIs using the concept of additive models. Additive models were proposed by Friedman and Stuetzle (Friedman and Stuetzle, 1981) and further developed and popularized by Stone (Stone, 1985), Hastie, and Tibshirani (Hastie and Tibshirani, 1990). Consider the regression problem where the feature lies in ${ℛ}^{d}$ and the objective function has a real value. Let $s_{1},\ldots ,s_{l}$ be a disjoint partition of the index set  {1,…,d} and denotes the elements of $s_{i}$ to be $j_{i1},\ldots ,j_{id_{i}}$ and $\pi_{i}\left(x\right)=\left(x_{j_{i1}},..,x_{j_{id_{i}}}\right)\in {ℛ}^{d_{i}}$ . Now, real-valued function F on ${ℛ}^{d}$ is said to be additive for partition { $s_{1},\ldots ,s_{l}$ } if there exists $F_{i}:\;{ℛ}^{d_{i}}\to ℛ$ such that

$$F\left(x\right)=\;\sum_{i=1}^{l}F_{i}(\pi_{i}\left(x\right)).$$ (2)

In our setting, the samples are elements in ${\left\{0,1,2\right\}}^{p+q}\subset {ℛ}^{p+q}$ , and we let $s_{1}=\left\{1,2,\ldots ,p\right\}$ and $s_{2}=\left\{1,2,\ldots ,q\right\}$ . We defined the absence of interaction between the two genes as the log odds ratio being additive with respect to the partition { $s_{1},s_{2}$ }. In other words, our null hypothesis is

$$H_{0}:\;\exists \;F_{1},F_{2}\;\text{such that}\;\;P\left(y=1\text{|}G_{1},G_{2}\right)=\;\frac{\text{exp}\left(F_{1}\left(G_{1}\right)+F_{2}\left(G_{2}\right)\right)}{1+\;\text{exp}\left(F_{1}\left(G_{1}\right)+F_{2}\left(G_{2}\right)\right)}.$$ (3)

#### 2.1.3 eXtreme Gradient Boosting (XGBoost)

eXtreme Gradient Boosting (XGBoost) (Chen and Guestrin, 2016) is a scalable machine-learning system for tree boosting, which researchers apply to bioactive molecular prediction (Babajide Mustapha and Saeed, 2016), protein submitochondrial localization prediction (Yu et al., 2020a), miRNA-disease association prediction (Chen et al., 2018), and in the bioinformatics field (Shao et al., 2021).

For a given dataset with n samples, $D=\left\{\left(x_{i},y_{i}\right)\right\},\;x_{i}\in {ℛ}^{m},y_{i}\in ℛ$ , and the XGBoost objective function is defined as:

$$obj\left(\theta \right)=\sum_{i}^{n}l\left(y_{i},\hat{y_{i}}\right)+\sum_{t=1}^{T}\text{Ω}\left(f_{t}\right),$$ (4)

where l is the loss function and Ω is the regularizer on the regression tree $f_{t}$ , $\theta =\left(f_{1},\ldots ,f_{T}\right)$ , and $\hat{y_{i}}=\sum_{t}f_{t}\left(x_{i}\right)\;$ . The t−th tree $f_{t}$ was obtained iteratively by gradient boosting, that is,

$$f_{t}\approx \arg\min\sum_{i}\left(\partial_{{\hat{y}}^{\left(t-1\right)}}l\left(y_{i},{\hat{y}}^{\left(t-1\right)}\right)f_{t}\left(x_{i}\right)+\frac{1}{2}\partial_{{\hat{y}}^{\left(t-1\right)}}^{2}l\left(y_{i},{\hat{y}}^{\left(t-1\right)}\right)f_{t}{\;}^{2}\left(x_{i}\right)\right)+\text{Ω}\left(f_{t}\right).$$ (5)

In our setting,

$$l\left(y_{i},\hat{y_{i}}\right)=\{\begin{array}{c} -\log\left(\frac{\exp\left(\hat{y_{i}}\right)}{1+\exp\left(\hat{y_{i}}\right)}\right)\;\;\;\;\;y=1\\ -\log\left(\frac{1}{1+\exp\left(\hat{y_{i}}\right)}\right)\;\;\;\;y=0 \end{array}\;\;\;\;\;$$ (6)

When running XGBoost, an essential step is to optimize its general parameters, booster parameters, and learning parameters.

#### 2.1.4 XGBoost With the Additive Constraint

**FIGURE 1:**
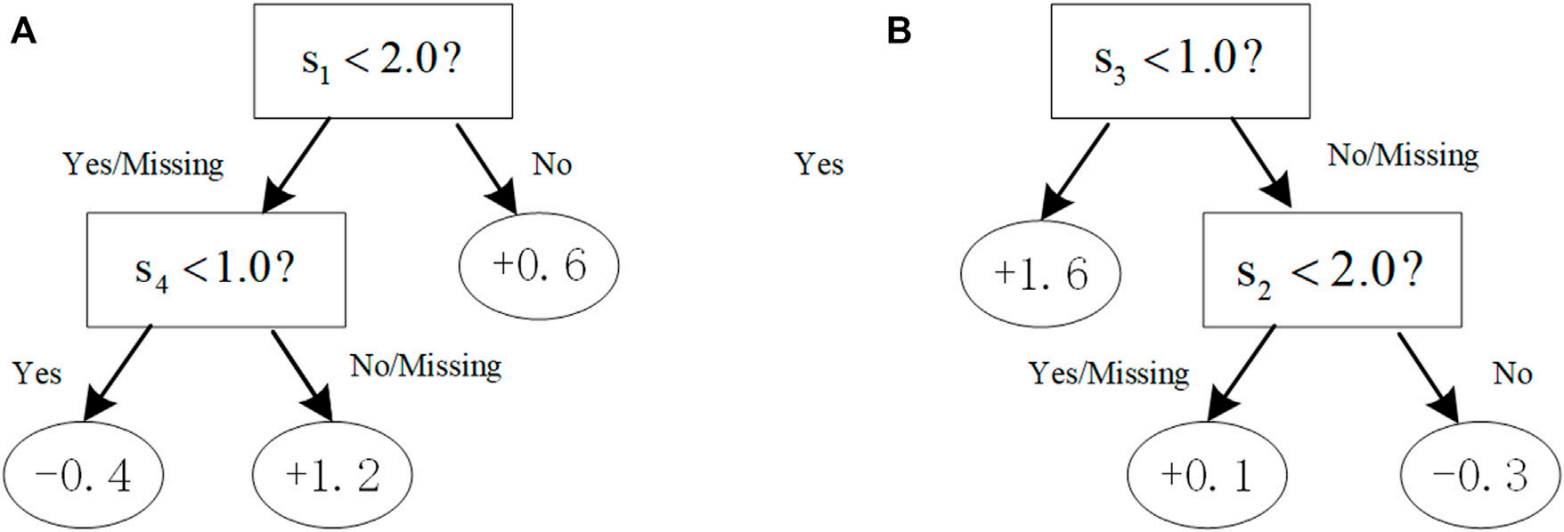
Illustration of trees with the additive constraint. With the constraint [[0,1] (Hindorff et al., 2009; Liu et al., 2018a; Loos, 2020),], **(A)** a tree violates the first constraint (0,1) that would not be in the boosting tree system; **(B)** a tree complies with both the first and second constraints.

The base learner $f_{t}$ used in XGBoost is a regression tree, and we considered features that appear in a path on the tree that starts at the root and ends at one of the leaves as features that interact with one another. XGBoost allows specification of feature interaction constraints in the form of lists of features where only the features in the same list are allowed to interact with one another. It is evident that when the lists are disjointed, and if every feature is included in one of the lists, the feature interaction constraint is equivalent to forcing each $f_{t}$ to include only features in a single list, which implies that the regression model $\sum_{i}f_{i}$ must be additive concerning the partition specified by the lists. With the constraint [[0,1] (Hindorff et al., 2009; Liu et al., 2018a; Loos, 2020),], for example, the tree in Figure 1A violates the first constraint [0,1], thus so would not be in the boosting tree system, but the tree in Figure 1B complies with both the first and second constraints.

#### 2.1.5 Illustration of the GGInt-XGBoost Workflow

Assume there are n samples in a case–control study for a pair of genes such that $G_{1}$ has  p SNPs and $G_{2}$ has q SNPs. We can then apply XGBoost using the logistic regression loss function with and without constraints on additivity, to the dataset to estimate the performance in error using 10-fold cross-validation. We denote the error in the unconstrained model as $err_{orig}^{0}$ and in the constrained model as $err_{cons}^{0}$ . The improvement of the performance of the unconstrained model over the constrained model is $\Delta err^{0}=\frac{err_{cons}^{0}-err_{orig}^{0}}{err_{orig}^{0}}$ , which according to our assumption should be a statistic that characterizes the strength of interaction between these two genes. A positive $\Delta err^{0}$ indicated that the unconstrained model performed better, and a larger positive $\Delta err^{0}$ means there was a stronger interaction between the two genes.

To get a *p*-value, we needed to estimate the distribution of $\Delta err^{0}$ under the null hypothesis. Here, we used a non-parametric strategy based on permutation: we shuffled the label y randomly m times, calculated Δerr using the exact same aforementioned procedure, and used the resulting empirical distribution as an estimate for the distribution of $\Delta err^{0}$ under the null hypothesis. Let the result of these m permutations be $\Delta err^{1},\ldots ,\Delta err^{m}$ , then an estimated *p*-value for the null hypothesis is

$$p=\frac{\left|\left\{i:\Delta err^{i}\geq \Delta err^{0}\right\}\right|}{m}.$$ (7)

For XGBoost, if we have n samples, K trees, a maximum depth of d per tree, and ||s||  as the number of non-missing entries in the training data, the training time complexity is O(Kd||s||logn) . Prediction for a new sample takes O(kd) . We employed parallel programming to minimize the execution time of permutation resampling.

**FIGURE 2:**
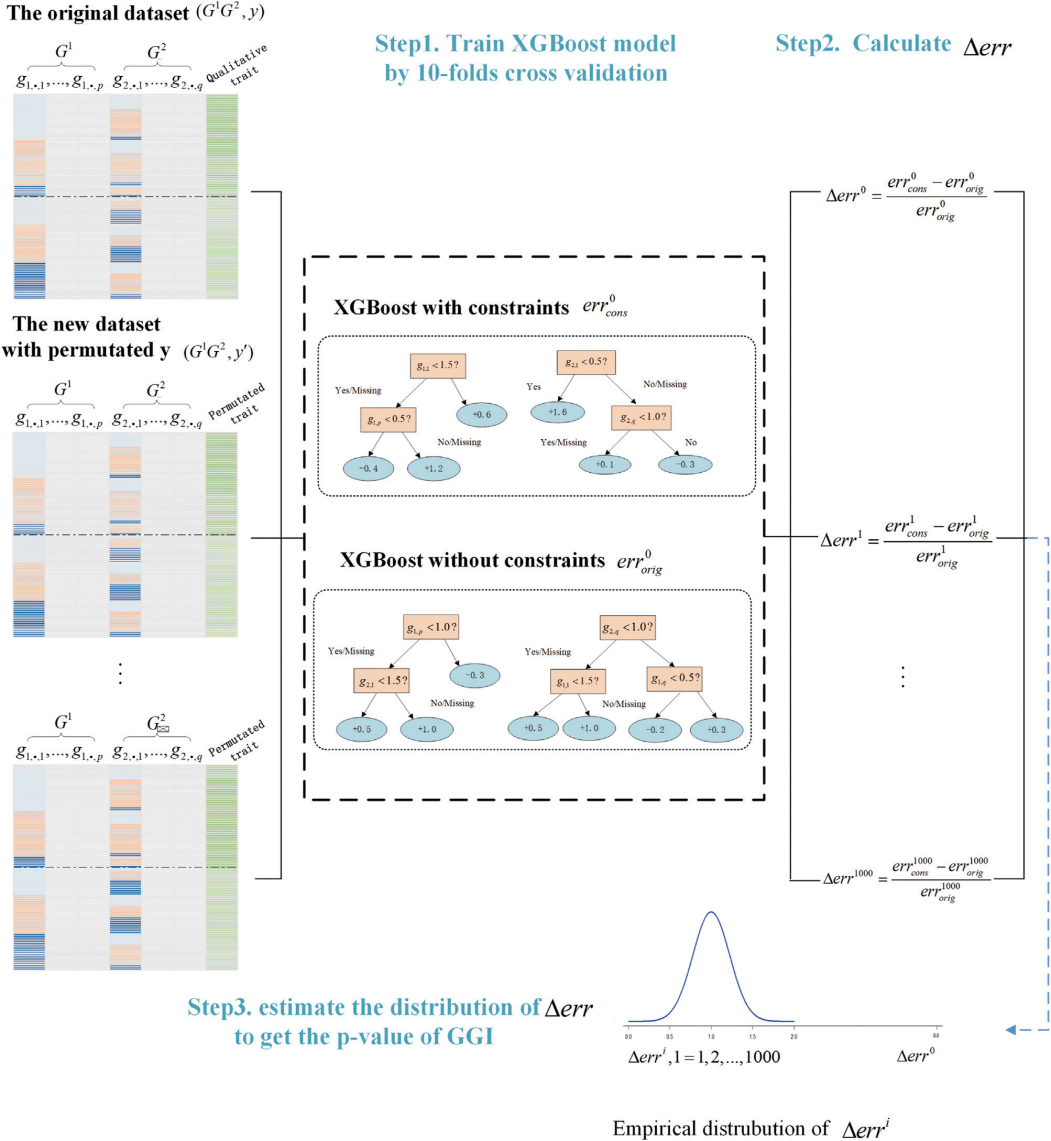
Illustration of the GGInt-XGBoost workflow for gene-based gene–gene interaction detection.


We summarized the process of GGInt-XGBoost in the algorithm below (Algorithm 1) and presented the overall workflow (Figure 2).

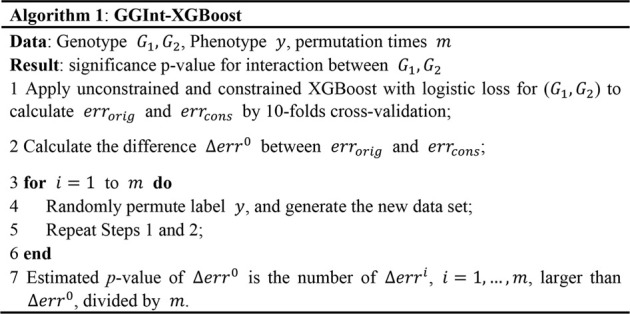


### 2.2 Simulation Study

To assess the performance of GGInt-XGBoost to control type I error and to detect GGIs, we compared GGInt-XGBoost with KCCA (Larson et al., 2013), GBIGM (Li et al., 2015b), and AGGrEGATOr (Emily, 2016).

#### 2.2.1 Simulation With Haplotype Data

**FIGURE 3:**
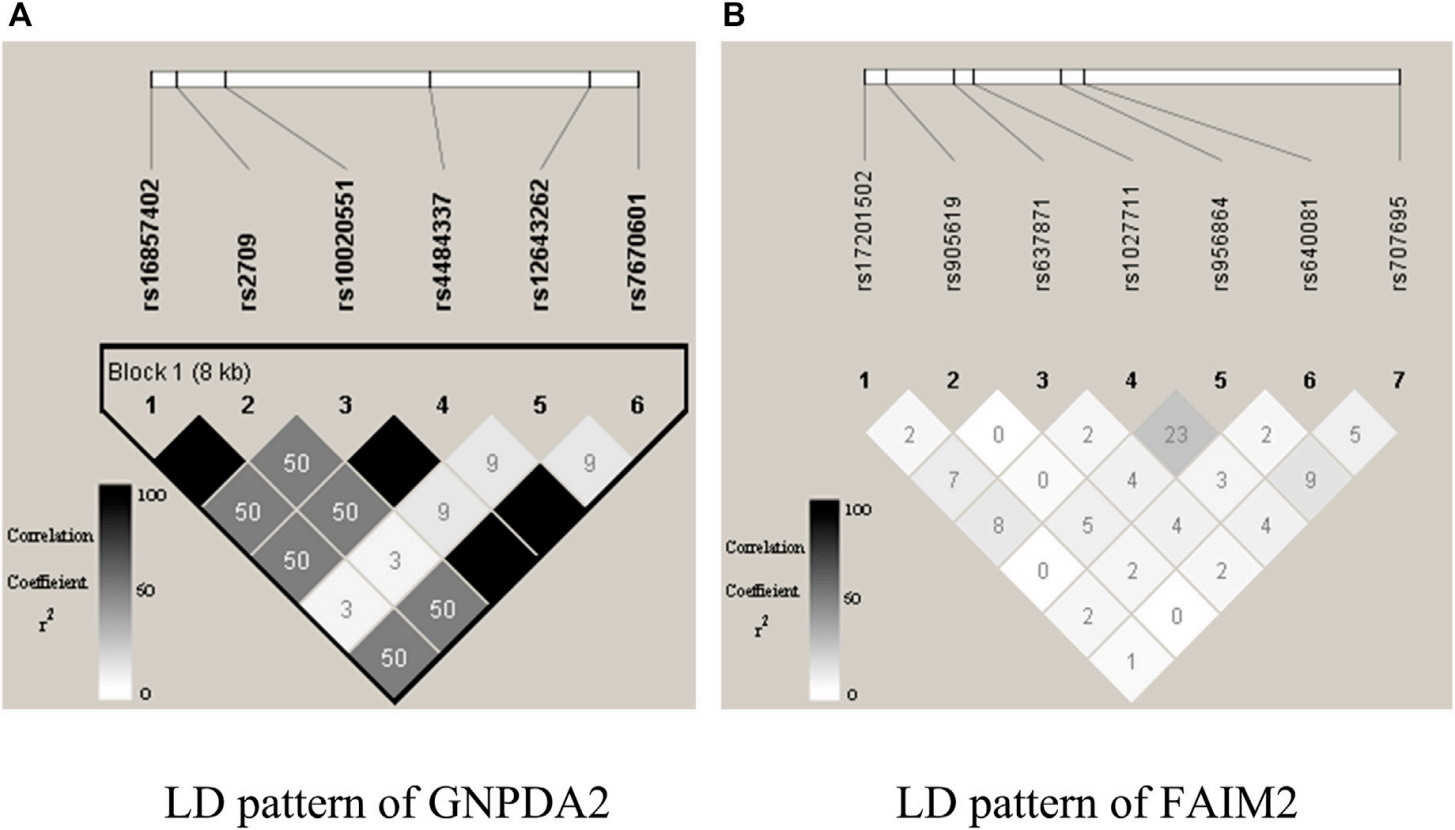
Illustration of LD structures within genes GNPDA2 and FAIM2. The plots are generated by Haploview. $r^{2}$ measures the LD strength of each pair of SNPs in each square, $0\leq r^{2}\leq 1$ , where 0 indicates no LD and 1 indicates complete LD. The GNPDA2 has a much stronger LD pattern than that within FAIM2, and they are not correlated.

gs2.0 (Li and Chen, 2008) is a semi-empirical simulation data generator that employs haplotype data as input and produces high-density SNP genotype data for qualitative samples. The generated dataset shares the same local linkage disequilibrium (LD) structure as that of human populations. We selected HapMap3 (a resident of Utah, the United States with Northern and Western European ancestry from https://www.sanger.ac.uk/resources/downloads/human/hapmap3.html) to mimic the actual LD structure of the human population. The Central European (CEU) dataset with 90 haplotypes was used as the template haplotype data. In this research, we randomly picked one pair of gene loci (i.e., GNPDA2 from chromosome 4 and FAIM2 from chromosome 12). GNPDA2 had a much stronger LD pattern than FAIM2 did, and they were not correlated (Figure 3). By employing the genipe module (Lemieux Perreault et al., 2016), an imputation pipeline on the genome-scale with PLINK, IMPUTE 2, and SHAPEIT, chromosomes 4 and 12 were imputed. After imputing, six SNPs were obtained from GNPDA2, and seven SNPs were obtained from FAIM2 (Supplementary Table S1).

#### 2.2.2 Disease Model

Here, we generated a disease model with two loci. A disease model represents the relationship between two loci that correspond to the disease. With various combinations of odds ratios (OR), sample sizes, and population prevalence, we generated different disease models. Using the jointly recessive–dominant model (RD model) as an example, for each locus let the genotype OR be (1+θ) and the population prevalence of the disease be p (Supplementary Table S2).

$\mathrm{Pr}\left(D\text{|}g_{i}\right)$ indicates the probability of a sample being a case given the genotype combination of $g_{i}$ and named the penetrance of $g_{i}$ , and $\mathrm{Pr}\left(\bar{D}\text{|}g_{i}\right)$ denotes the probability of a sample being a control given the genotype combination of $g_{i}$ . Then, the odds of disease are:

$$ODD_{g_{i}}=\frac{\mathrm{Pr}\left(D\text{|}g_{i}\right)}{\mathrm{Pr}\left(\bar{D}\text{|}g_{i}\right)}=\frac{\mathrm{Pr}\left(D\text{|}g_{i}\right)}{1-\mathrm{Pr}\left(D\text{|}g_{i}\right)}.$$ (8)

The penetrance of genotype $g_{i}$ can be calculated using:

$$\mathrm{Pr}\left(g_{i}\right)=\frac{ODD_{g_{i}}}{1+ODD_{g_{i}}}.$$ (9)

The corresponding penetrance table is shown in Supplementary Table S3.

With a specific genotype OR (1+θ) and a population prevalence p , the baseline value γ represents the disease odds with the two loci that do not have the disease alleles, and it can be calculated by applying Eq. 10 with the terms from Supplementary Table S3.

$$p=Pr\left(D\right)=\sum \mathrm{Pr}\left(D\text{|}g_{i}\right)\times \text{Pr}\left(g_{i}\right).$$ (10)

We used six integrated disease models in gs2.0, which included a recessive–dominant model, a dominant–dominant model, an XOR model, a threshold model, a multiplicative model, and a recessive–recessive model. We generated different datasets by various parameter settings, and we compared the performances of KCCU, GBIGM, and AGGrEGATOr with our method.

*Evaluation of Type-I Error*: The type-I error indicates the ability of a method to reject the null hypothesis when it is true. In this study, the significance level α  was set to 0.05. The simulation used in the model is shown in Supplementary Table S4 and run 100 times with each sample size n∈{1k,2k,3k,4k,5k} with the odds ratio set at 1.

*Evaluation of Power of the Test*: The power of a test indicates the probability that the method can reject the null hypothesis correctly when the alternative hypothesis is true. In this study, we generated 100 datasets for each parameter set under six disease models (Supplementary Table S5). The power under each parameter setting was expressed by the frequency with which the null hypothesis of the dataset was rejected correctly at the significance level of α=0.05 . To evaluate the influence of sample size, we chose n∈{1k,2k,3k,4k,5k} with a specific population prevalence p=0.01 and OR=2 . To assess the impact of the OR, we considered varying OR∈{1.5,2,2.5,3,3.5,4} given a sample size of n=4000 (cases and controls were both 2000, balanced dataset) and p=0.01 .

For GGInt-XGBoost, KCCU, AGGrEGATOr, and GBIGM, if the number of datasets with a significance level less than α  is $m_{1}$ , then the power can be calculated by the following formula:

$$power=\;\frac{m_{1}}{100}.$$ (11)

GBIGM and AGGrEGATOr methods are nonparametric methods so no parameters need to be specific. We only set the ratio of the trimmed jackknife to 0.05 ( ω=0.05 ) for KCCU. For GGInt-XGBoost, we set the number of trees to 1,000 (num_round = 1,000), the maximum depth of trees to 3 (max_depth = 3), the type of objective to “binary:logistic” (objective = “binary:logistic”), the learning rate to 0.01 (eta = 0.01), and the evaluate metric to error (eval_metric = “error”). We recommend that when dealing with real-data analysis, the depth of the trees is not to be set too deep in order to avoid overfitting. For a dataset with thousands of samples, a maximum depth of 2–4 is usually sufficient.

### 2.3 Experiments Using Rheumatoid Arthritis Data

To evaluate GGInt-XGBoost’s ability to process real GGIs in a qualitative dataset, we analyzed the susceptibility of a series of pairs of genes in rheumatoid arthritis (RA), a chronic systemic disease with inflammatory synovitis with unknown etiology. It causes progressive bone destruction and affects bone remodeling. In this article, we chose the WTCCC (2007) dataset, which contained British population genotype data generated by the Affymetrix GeneChip 500 k. We preprocessed our dataset in the following ways:

i. To verify the GGIs in the RA, we selected the pathway hsa05323 from the KEGG pathway database. The genotyping coordinates of the WTCCC dataset can be found in UCSC hg18/NCBI Build36. There were 90 genes in this pathway. Among them, many genes belonged to MHCII and V-ATPase, which are two protein combinations. Because many GGIs occurred by themselves, we only selected representative genes from each protein combination, and then we excluded other genes. Finally, 48 genes remained, which resulted in $C_{48}^{2}=1128$ pairs of genes to be evaluated.

ii. The detailed gene information was obtained from the annotation file of NCBI Build36. For each gene, we added a 10 kb buffer region both downstream and upstream of the originally defined gene position. All SNPs within the region were selected for each gene.

iii. According to the quality control of GWAS, samples that included gender that did not match the chromosome X heterozygote rates were removed. SNPs were also excluded when they met any of the following conditions: the missing rate in the sample was ≥10% , the minor allele frequency (MAF) was ≤0.05 , or the frequency of the control violated the Hardy–Weinberg equilibrium ( p<0.0001 ). Finally, 385 SNPs remained with 4,966 samples that consisted of 2,993 control subjects and 1973 case subjects.

## 3 Results and Discussion

The experimental environment of the following results was a workstation with an Intel Xeon CPU E5-2,620 v2 at 2.10GHz, 96 GB of DDR3, Python3.6, and RStudio programming implementation.

### 3.1 Simulation Study

#### 3.1.1 Evaluation of Type-I Error

For type-I error, by setting the significance level at α=0.05 , we varied the sample size from 1,000 to 5,000. All the methods tested, except for GBIGM when n=1,000 , had a type-I error comparable to the significance level (Table 1), which implied that these methods well controlled type-I error for various sample sizes.

**TABLE 1:** Type-I error for KCCU, GBIGM, AGGrEGATOr, and GGInt-XGBoost when varying the sample size.

Method	Sample size				
	1,000	2,000	3 *,*000	4,000	5,000
KCCU	0.02	0.02	0.01	0.05	0.07
GBIGM	0.13	0.06	0.07	0.07	0.07
AGGrEGATOr	0.05	0.06	0.07	0.04	0.02
GGInt-XGBoost	0.03	0.06	0.07	0.04	0.06

#### 3.1.2 Evaluation of the Power of the Test

##### Impact of Odds Ratio

**FIGURE 4:**
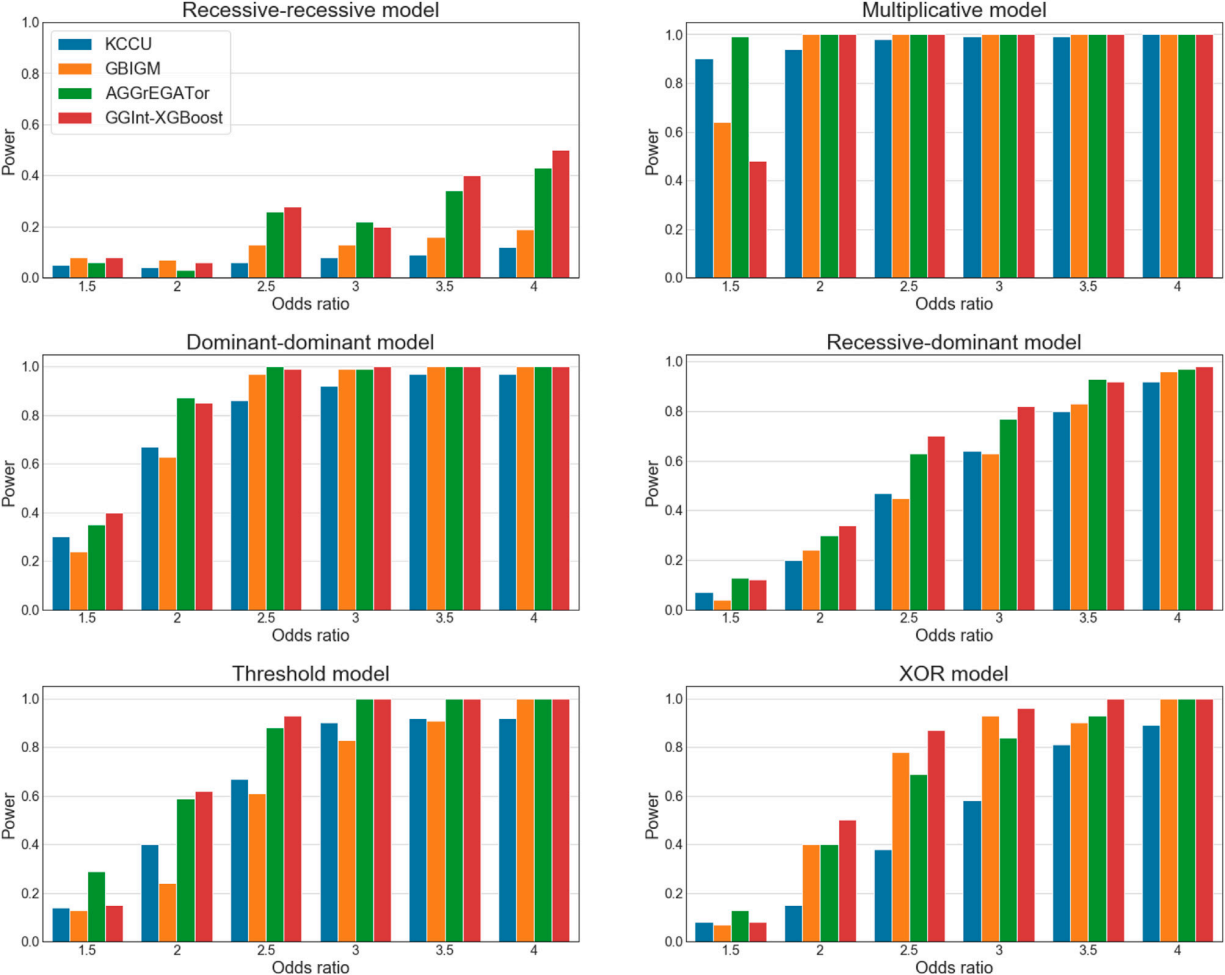
Statistical power of simulation studies for KCCU (blue), GBIGM (yellow), AGGrEGATOr (green), and GGInt-XGBoost (red) under six disease models with OR∈{1.5,2,2.5,3,3.5,4}

We investigated the performance of the various methods in detecting GGIs under the six disease models, with a population prevalence of 0.01 , the sample size of 4,000 , and odds ratios that varied from 1.5 to 4 (Figure 4). For this experiment, a single pair of SNPs that belonged to different genes was chosen randomly for the disease models in the generation of the simulated dataset, and the genes that contained these two SNPs were considered to be interacting. A larger OR resulted in better performance for all methods, and, when OR=4 , some methods had a power that approached 1 (Figure 4). Our method was the best among all methods tested except for when OR=1.5 , which might be because the base learner of XGBoost was a regression tree that might be prone to overfitting. It would be difficult to distinguish the signal from noise when the interaction strength was too low.

It is worth noting that in the recessive–recessive model (RR model) (Supplementary Table S5A), the detection power was consistently ≤20% as the OR value changed gradually from 1.5 to 4. AGGrEGATOr and GGInt-XGBoost reached approximately 45%. According to the RR model penetrance table, the baseline γ was very small when the population prevalence was p=0.01 . Therefore, of the nine genotype combinations, eight of them tended to be zero. The only causal genotype (aabb) contained two minor alleles. Typically, the MAF of a SNP ranged from 0.2 to 0.4, and few genotypes (aabb) appeared in the simulation dataset. Consequently, it was difficult to detect the GGI under the disease phenotype. This was the main reason for the poor performance of these methods under the RR model.

##### Impact of Sample Size

We also investigated the influence of the sample size. Let the sample size be n∈{1k,2k,3k,4k,5k} , p=0.01 , and OR=2 (Supplementary Figure S1). As the sample size increased, the detection power of all methods increased monotonically under all disease models, except for the RR model. In all methods, a larger sample size corresponded to better performance.

In conclusion, GGInt-XGBoost performed better in simulation studies than the other methods tested in almost every setting. The reason was probably that our method, by making use of constrained and unconstrained XGBoost models, made weak assumptions on the kind of interaction because any deviation from the additivity in the prediction of log odds ratios indicated an underlying GGI, which resulted in better statistical power. Furthermore, our method was more robust with respect to the LD pattern among the SNPs within each gene because the additivity constraint did not destroy the LD structure within each gene.

### 3.2 Experiments Using Rheumatoid Arthritis Data

Rheumatoid arthritis (RA) is an autoimmune disease with symptoms that typically include pannus formation in the synovial joints and destruction of cartilage and bones. The genes IL-17, IL-6, TNF−α , RANK, and MMP3 are related to the development of RA (Majithia and Geraci, 2007). There were 48 genes in our dataset chosen from the RA pathway hsa05323, which resulted in 1,128 pairs of genes. We set significance level to α=0.01 , and for our method, the number of permutations were set to m=1000 . GBIGM and KCCU resulted in 134 and 159 pairs of detected interacting genes, respectively. A total number of 65 of those pairs that were detected by GBIGM and 30 of the pairs that KCCU detected had a *p*-value of 0 . AGGrEGATOr detected 17 pairs of interacting genes, and GGInt-XGBoost detected 58 pairs of interacting genes.

Because there were too many detected interacting gene pairs in KCCU and GBIGM with a *p*-value = 0, we could not analyze all of them in detail, so we focused on the 10 most significant gene pairs detected by AGGrEGATOr and, by our method, GGInt-XGBoost (Table 2). After a literature search, we found 7 of the 10 most significant gene pairs under GGInt-XGBoost and 3 of the 10 most significant gene pairs under AGGrEGATOr were supported by prior research. There was also a greater correlation between the results of GGInt-XGBoost and KCCU or GBIGM than the correlation between AGGrEGATOr and GBIGM or KCCU.

**TABLE 2:** Calculated *p*-value for the 20 gene pairs using all four different methods. *p*-values in bold font indicate that they are significant. The ``Ref'' column indicates that the pair can be found as direct interaction in our literature search.

Gene1	Gene2	Ref	*p*-value			
			GGInt-XGBoost	AGGrEGATOr	KCCU	GBIGM
HLA class II	TGF β	Navarrete Santos et al. (1998)	**0.0**	0.588	**0.0**	**0.037**
HLA class II	LFA-1	Steere and Glickstein, (2004)	**0.0**	0.591	0.195	0.373
HLA class II	TEK		**0.0**	0.213	0.521	0.226
IL-8	ANG-1	Kabala et al. (2020)	**0.0**	1.0	0.818	0.32
MMP-3	April		**0.0**	0.164	0.161	0.063
HLA-DQA1	ANG-1		**0.0**	1.0	0.788	0.962
CTLA4	HLA class II	Karlson et al. (2008)	**0.0**	0.663	0.292	**0.0**
MMP-3	BLYs	Narasimhan et al. (2018)	**0.0**	0.473	**0.0001**	0.5
JUN	FOS	Huber et al. (2019)	**0.0**	0.441	0.692	**0.025**
GM-CSF	HLA class II	Field, (1995)	**0.0**	0.391	**0.047**	**0.0**
CD80	April		0.549	**0.0006**	0.941	0.334
CTSK	BLYS		**0.0**	**0.0008**	0.356	0.056
AP-1	IL-6		0.764	**0.0018**	0.098	0.287
CD86	CTSL		0.235	**0.0019**	0.519	0.252
CXCL6	FLT-1		0.098	**0.0023**	**0.004**	0.52
CTLA4	AP-1	Schneider and Rudd, (2014)	0.843	**0.0023**	**0.042**	0.102
FLT-1	LFA-1		0.117	**0.0031**	0.063	**0.028**
CCL3	TRAP	Jordan et al. (2018)	0.098	**0.0032**	0.682	**0**
IL-18	TGF β		0.137	**0.0036**	0.149	0.22
IL-1	SDF-1		0.647	**0.004**	0.116	0.636

Furthermore, when using GGInt-XGBoost, after the detection of interacting gene pairs, one can also use the ensemble tree mechanism of XGBoost to investigate marker-based interactions further; this is because the regression tree, which is the base learner used in XGBoost, is a powerful tool for the discovery of interactions among features. For a regression tree model, one considers features that appear in the same traversal path from the root to leaf to be interacting. As an example, the gene pairs IL-8 and Ang-1 were found to interact using our method. Pawel et al.(Kabala et al., 2020) reported that Ang-1 induced the production of IL-8 in synovial tissue explants of RA patients. In the first tree in the unconstrained XGBoost model, it was clear that one SNP from the gene IL-8 on chromosome 4 interacted with rs121937926 in the gene Ang-1 (Supplementary Figure S2). The interaction form was flexible because our method imposed no functional form.

**FIGURE 5:**
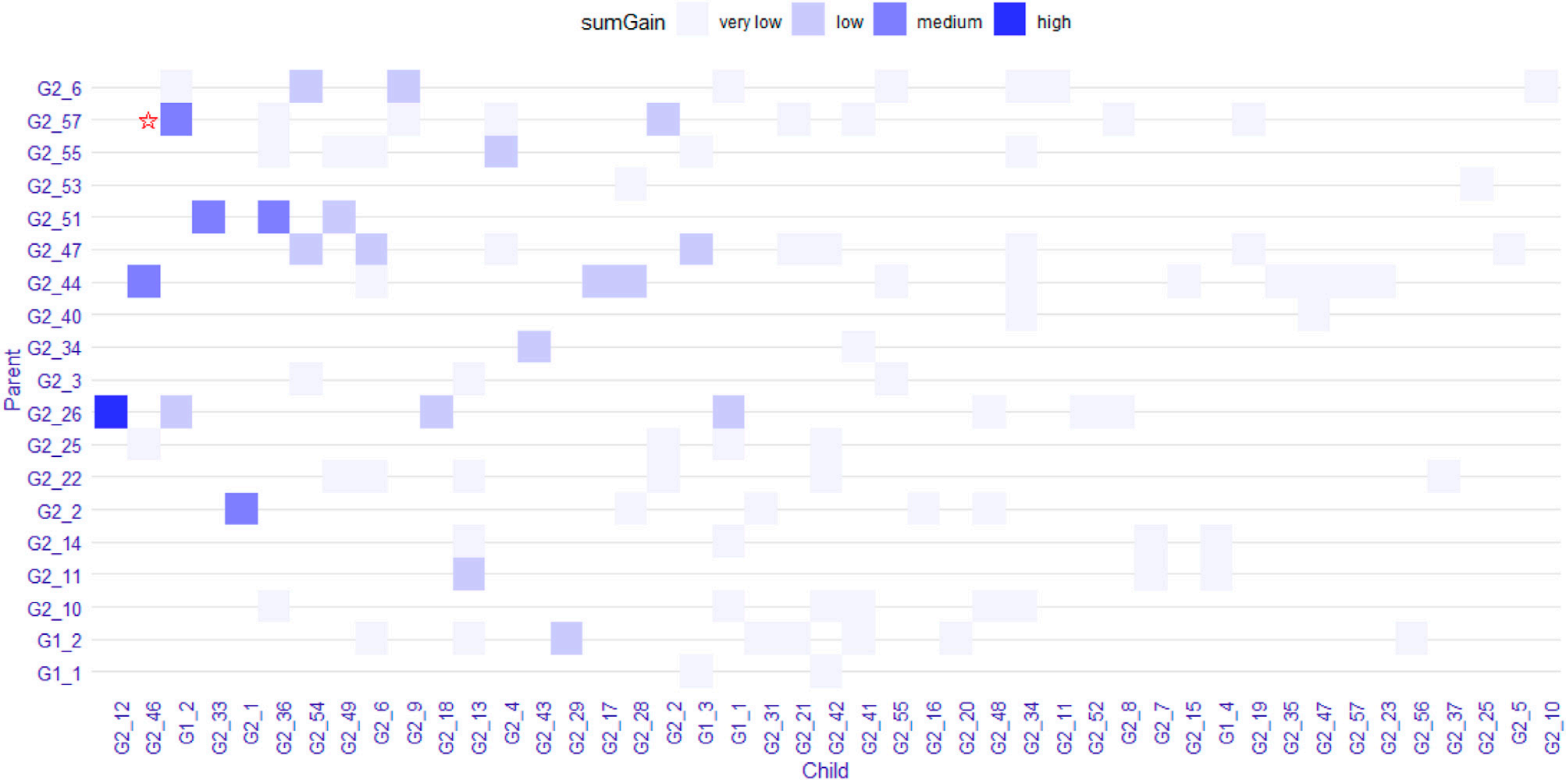
Plot that shows pairs of SNPs that lie in two nodes of a regression tree connected by an edge. The color indicates the sumGain measure for the SNP pairs. The pair with the red star indicates the interacting SNP pair from IL-8 and Ang-I.

We explored the structure of the unconstrained XGBoost model further with R package EIX (Karbowiak and Biecek, 2021), which produced an interaction plot (Figure 5). For the convenience of display, all the SNPs in IL-8 were named “G1_X”, and all SNPs in ANG-1 were named “G2_X”, where “X” was the index. We chose the sumGain as a measure for the interaction strength. The sumGain was the sum of the gain value in all nodes in which the given SNP occurred. The intensity of the sumGain was divided into four equal parts and represented by different colored squares in the legend. The interacting SNP pairs in Supplementary Figure S2 from IL-8 and Ang-1 exhibited median strength in Figure 5 (with red star), which demonstrated that it was possible to use the results of GGInt-XGBoost for a more fine-grained analysis of GGIs at the marker level. Also, our method was robust with respect to LD because the LD structure within each gene was still expressed in the tree model and did not directly impact the performance of our method (Figure 5). Table 3 gives the information of the top 10 interacted SNP pairs by sumGain and occurrence frequency in the ensemble boosting trees.

**TABLE 3:** sumGain measure of the 10 most significant interacting SNP pairs from IL-8 and Ang-I. “Frequency” is the number of occurrences of the SNP pair in the trained model.

Index	Parent_SNP	Child_SNP	sumGain	Frequency
1	G2_26	G2_12	387.846,816	87
2	G2_44	G2_46	237.672,225	50
3	G2_57	G1_2	228.947,974	75
4	G2_51	G2_33	218.003046	33
5	G2_2	G2_1	214.650,794	68
6	G2_51	G2_36	213.414,267	28
7	G2_6	G2_54	184.671,309	81
8	G2_51	G2_49	178.749,247	27
9	G2_47	G2_6	154.492,033	31
10	G2_6	G2_9	140.040435	54

## 4 Conclusion

Gene–gene interactions (GGIs) are important in the study of complex diseases and traits. In this article, we developed a gene-based GGI detection algorithm called GGInt-XGBoost. We treated the GGI detection problem as a measure of how much the log odds ratio of qualitative traits deviated from the additive structure. GGInt-XGBoost benefits from the attractive built-in mechanism of XGBoost that allows for an elegant expression of the additive structure by adding feature interaction constraints. Because of the weak assumptions of the interaction form and powerful and practical ability of XGBoost to fit nonlinear relationships, our method detected more types of interpretable GGIs accurately and effectively than other methods.

Combined with logistic regression, GGInt-XGBoost can be used for the GWAS of complex qualitative traits. To test its performance, we conducted a semi-empirical simulation study and a retrospective analysis of rheumatoid arthritis. For most of the settings tested, GGInt-XGBoost outperformed prior methods in statistical power for detecting GGIs. Also, because the base learner we used in XGBoost was the regression tree, GGIng-XGBoost can detect GGIs under quantitative traits. Furthermore, the base learner of XGBoost is a tree model that has a natural way of expressing marker-based interactions, which allows further investigations of interactions at the marker level after two genes are known to interact. For example, we looked for interactions between the genes IL-8 and Ang-1 and found that it was largely accounted for by the interaction between a single pair of SNPs from these two genes. Also, through the analysis of IL-8 and Ang-1, we found that GGInt-XGBoost was robust with respect to the LD structure within genes. The workflow designed for detection of GGIs did not damage the LD structure, and the assumption of the additive structure allowed marker-based interaction within genes. Last, GGInt-XGBoost might be improved further or generalized by incorporating ideas from causal inferences that would be applied more effectively to multi-gene settings and the study of gene pathways. In conclusion, GGInt-XGBoost is a helpful addition to the existing toolbox of statistical methods for studying gene–gene interaction in genome-wide association studies.

## Data Availability

Publicly available datasets were analyzed in this study. These data can be found here: https://www.wtccc.org.uk/info/access_to_data_samples.html
