# Supplementary material for: Gene-Based Testing of Interactions Using XGBoost in Genome-Wide Association Studies
Source: Front Cell Dev Biol. 2021 Dec 16;9:801113. doi: 10.3389/fcell.2021.801113 (PMC8716787; doi:10.3389/fcell.2021.801113)
Supplement: Supplementary file 1 [file DataSheet1.docx]

**Supplementary materials for the manuscript**

**“Gene-based testing of interactions using XGBoost in genome-wide association studies”**

Yingjie Guo^1,2^, Chenxi Wu^3^, Zhian Yuan^4^, Yansu Wang^1,2^, Zhen Liang^5^, Yang Wang^2^, Yi Zhang^6*^ and Lei Xu^2*^

1. Institute of Fundamental and Frontier Sciences, University of Electronic Science and Technology of China, No.4 Block 2 North Jianshe Road, Chengdu, 610054, China
2. School of Electronic and Communication Engineering, Shenzhen Polytechnic, 7098 Liuxian Street, Shenzhen, 518000, China
3. Department of Mathematics, University of Wisconsin-Madison, 480 Lincoln Dr., Madison, WI 53706, United States.
4. Research Institute of Big Data Science And Industry, Shanxi University, 92 Wucheng Road, Taiyuan, 030006，China;
5. School of Life Science, Shanxi University, 92 Wucheng Road, Taiyuan, 030006, China.
6. Beidahuang Industry Group General Hospital, Harbin, China

* Correspondence: [csleixu@szpt.edu.cn](mailto:csleixu@szpt.edu.cn), [y_zhang1024@126.com](mailto:y_zhang1024@126.com)

**Table S1**. The detailed information about rsID of each SNP in the dbSNP database and the physical position based on the GRCh37 build of genes GNPDA2 and FAIM2.

**Table S2**. The odds table for the RD model.

**Table S3**. The penetrance table for the RD model.

**Table S4**. Table of odds for type-I error model.

**Table S5**. Table of odds for six disease models with interaction between a pair of SNPs.

**Table S6**. NCBI-GeneID for genes in Table 2.

**Figure S1**. The empirical, simulation-based statistical power of KCCU (blue), GBIGM (yellow), AGGrEGATOr (green), and GGInt-XGBoost (red)

**Figure S2.** The illustration of the first tree structure in the XGBoost model.

**Table S1**. The detailed information about rsID of each SNP in the dbSNP database and the physical position based on the GRCh37 build of genes GNPDA2 and FAIM2. The column physical position is expressed by chr: base position.

| **Index** | | **Gene name** | **rsID** | **Physical- position** | | **Gene name** | | **rsID** | | **Physical- position** |  |
| --- | --- | --- | --- | --- | --- | --- | --- | --- | --- | --- | --- |
| 1 | GNPDA2 | | rs16857402 | | 4:44706453 | | FAIM2 | rs17201502 | 12: 50285562 | | |
| 2 |  |  | rs2709 | | 4:44706913 | |  | rs905619 | 12:50286055 | | |
| 3 |  |  | rs10020551 | | 4: 44707815 | |  | rs637871 | 12: 50287592 | | |
| 4 |  |  | rs4484337 | | 4: 44711547 | |  | rs1027711 | 12: 50288032 | | |
| 5 |  |  | rs12643262 | | 4: 44714455 | |  | rs956864 | 12: 50290023 | | |
| 6 |  |  | rs7670601 | | 4: 44715341 | |  | rs640081 | 12: 50290554 | | |
| 7 |  |  |  | |  | |  | rs707695 | 12: 50297670 | | |

**Table S2**. The odds table for the RD model, each cell indicates the odds of disease given that an individual has that genotype combination $g_{i}$.

| **SNP1** | **SNP2** | | |  |
| --- | --- | --- | --- | --- |
|  | **AA** | **Aa** | **aa** |  |
| **BB** | $\gamma$ | $\gamma$ | $\gamma$ |  |
| **Bb** | $\gamma$ | $\gamma$ | $\gamma(1+\theta)$ |  |
| **bb** | $\gamma$ | $\gamma$ | $\gamma(1+\theta)$ |  |

**Table S3**. The penetrance table for the RD model.

| **SNP1** | **SNP2** | | |
| --- | --- | --- | --- |
|  | **AA** | **Aa** | **aa** |
| **BB** | $\frac{\gamma}{1+\gamma}$ | $\frac{\gamma}{1+\gamma}$ | $\frac{\gamma}{1+\gamma}$ |
| **Bb** | $\frac{\gamma}{1+\gamma}$ | $\frac{\gamma}{1+\gamma}$ | $\frac{\gamma(1+\theta)}{1+\gamma(1+\theta)}$ |
| **bb** | $\frac{\gamma}{1+\gamma}$ | $\frac{\gamma}{1+\gamma}$ | $\frac{\gamma(1+\theta)}{1+\gamma(1+\theta)}$ |

**Table S4**. Table of odds for type-I error model, no effect model without epistasis between a pair of SNPs, $\gamma=1$.

|  | **AA** | **Aa** | **aa** |
| --- | --- | --- | --- |
| **BB** | $\gamma$ | $\gamma$ | $\gamma$ |
| **Bb** | $\gamma$ | $\gamma$ | $\gamma$ |
| **bb** | $\gamma$ | $\gamma$ | $\gamma$ |

**Table S5**. Table of odds for six disease models with interaction between a pair of SNPs

(a) Recessive-recessive (b) Interaction-multiplicative

| SNP1 | SNP2 | | |  | SNP2 | | |
| --- | --- | --- | --- | --- | --- | --- | --- |
|  | AA | Aa | aa |  | AA | Aa | aa |
| BB | $\gamma$ | $\gamma$ | $\gamma$ |  | $\gamma$ | $\gamma$ | $\gamma$ |
| Bb | $\gamma$ | $\gamma$ | $\gamma$ |  | $\gamma$ | $\gamma(1+\theta)$ | ${\gamma(1+\theta)}^{2}$ |
| bb | $\gamma$ | $\gamma$ | $\gamma(1+\theta)$ |  | $\gamma$ | ${\gamma(1+\theta)}^{2}$ | ${\gamma(1+\theta)}^{4}$ |

(c) XOR (d) Dominant-dominant

| SNP1 | SNP2 | | |  | SNP2 | | |
| --- | --- | --- | --- | --- | --- | --- | --- |
|  | AA | Aa | aa |  | AA | Aa | aa |
| BB | $\gamma$ | $\gamma$ | $\gamma(1+\theta)$ |  | $\gamma$ | $\gamma$ | $\gamma$ |
| Bb | $\gamma$ | $\gamma$ | $\gamma(1+\theta)$ |  | $\gamma$ | $\gamma(1+\theta)$ | $\gamma(1+\theta)$ |
| bb | $\gamma(1+\theta)$ | $\gamma(1+\theta)$ | $\gamma$ |  | $\gamma$ | $\gamma(1+\theta)$ | $\gamma(1+\theta)$ |

(e) Threshold (f) Recessive-dominant

| SNP1 | SNP2 | | |  | SNP2 | | |
| --- | --- | --- | --- | --- | --- | --- | --- |
|  | AA | Aa | aa |  | AA | Aa | aa |
| BB | $\gamma$ | $\gamma$ | $\gamma$ |  | $\gamma$ | $\gamma$ | $\gamma$ |
| Bb | $\gamma$ | $\gamma$ | $\gamma(1+\theta)$ |  | $\gamma$ | $\gamma$ | $\gamma(1+\theta)$ |
| bb | $\gamma$ | $\gamma(1+\theta)$ | $\gamma(1+\theta)$ |  | $\gamma$ | $\gamma$ | $\gamma(1+\theta)$ |

**Table S6**. NCBI-GeneID for genes in Table 2.

| Gene Name | NCBI-GeneID |
| --- | --- |
| HLA class II | 3117 |
| IL-8 | 3576 |
| MMP-3 | 4314 |
| HLA-DQA1 | 3117 |
| CTLA4 | 1493 |
| JUN | 3725 |
| GM-CSF | 1437 |
| CD80 | 941 |
| CTSK | 1513 |
| AP-1 | 2353 |
| CD86 | 942 |
| CXCL6 | 6372 |
| CTLA4 | 1493 |
| FLT-1 | 2321 |
| CCL3 | 6348 |
| IL-18 | 3606 |
| IL-1 | 3552 |
| TGF$\beta$ | 7043 |
| LFA-1 | 3689 |
| TEK | 7010 |
| ANG-1 | 284 |
| APRIL | 8741 |
| BLYs | 10673 |
| FOS | 2353 |
| IL-6 | 3569 |
| CTSL | 1514 |
| TRAP | 54 |
| SDF-1 | 6387 |


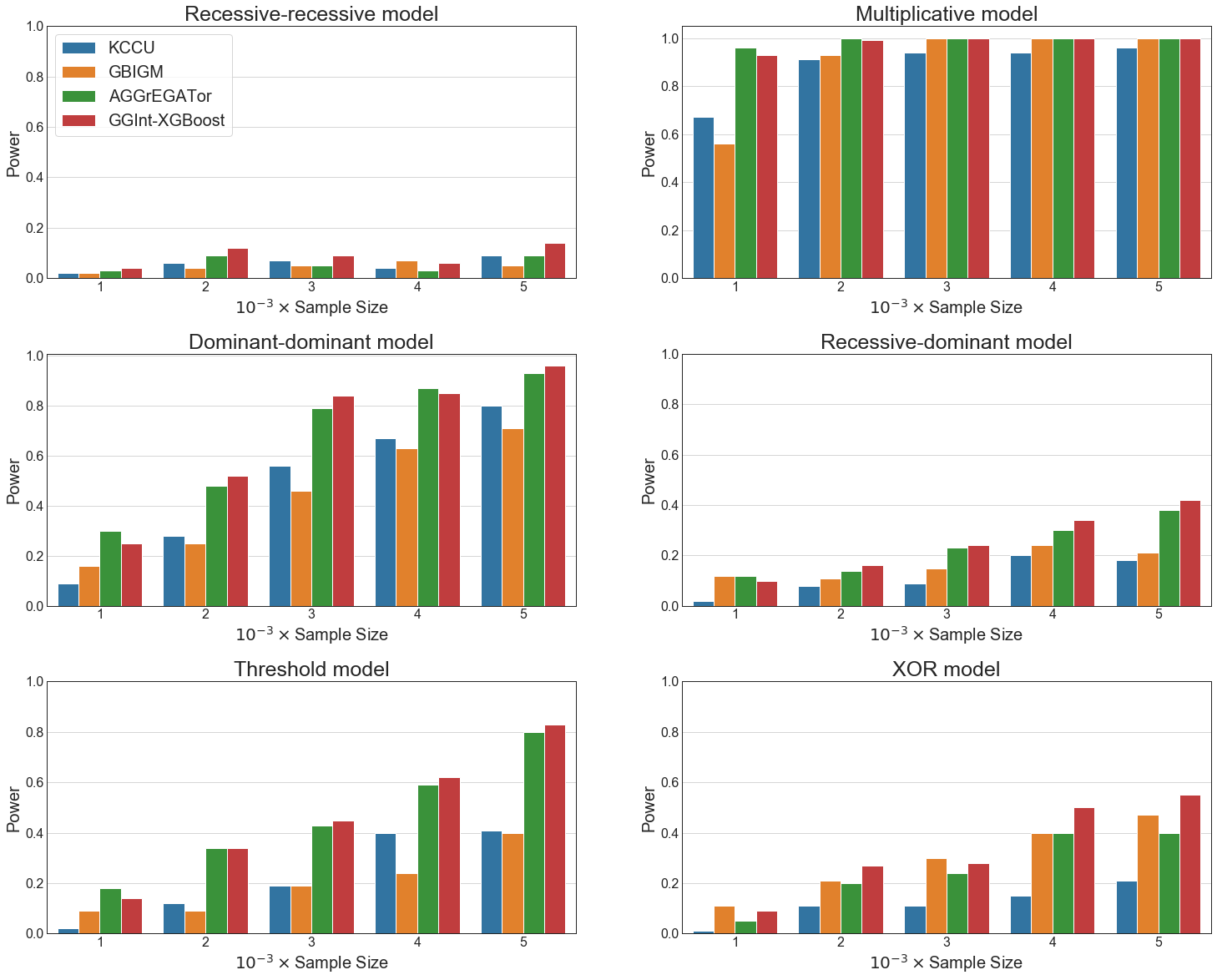


**Figure S1**. The empirical, simulation-based statistical power of KCCU (blue), GBIGM (yellow), AGGrEGATOr (green), and GGInt-XGBoost (red) under six disease models when varying the $n\in\{1k,2k,3k,4k,5k\}$


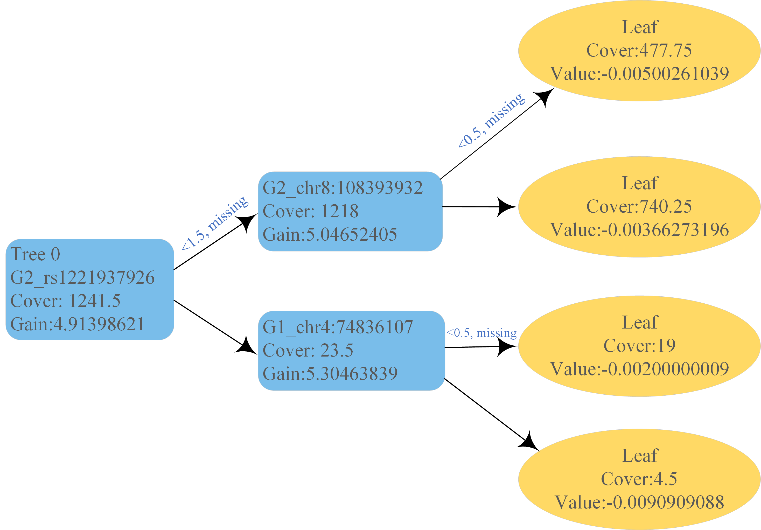


**Figure S2**. The illustration of the first tree structure in the XGBoost model. “G1” indicates the gene IL-8, and “G2” indicates the gene Ang-1. SNPs without rsID are denoted by chromosome number and base position.
